# Supplementary material for: The Thrombopoietin Receptor Agonist Eltrombopag Inhibits Human Cytomegalovirus Replication Via Iron Chelation
Source: Cells. 2019 Dec 20;9(1):31. doi: 10.3390/cells9010031 (PMC7017049; doi:10.3390/cells9010031)
Supplement: Supplementary file 1 [file cells-09-00031-s001.pdf]

Figure S1

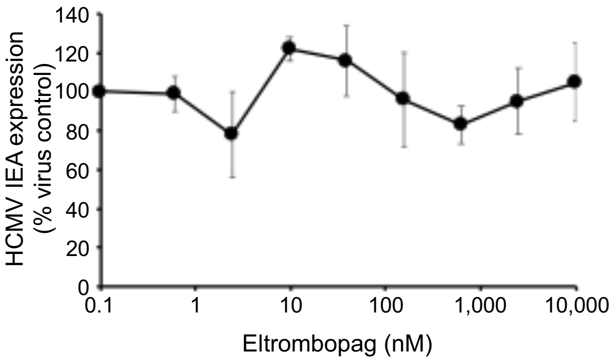

Figure 1. Effects of eltrombopag on HCMV immediate early antigen (IEA) expression in primary human foreskin fibroblasts (HFFs).

Table S1. Effects of eltrombopag addition at different time points during the HCMV replication cycle using human foreskin fibroblasts infected with HCMV strain Hi91 at an MOI of 0.02.

Time of drug addition	IC_{50}[1] (nM)
Continuous starting from virus infection	415 ± 197
24h pre-treatment	> 10
During the 1h virus adsorption period	8,979 ± 1153
1h post infection	233 ± 35
24h post infection	358 ± 105
48h post infection	2,493 ± 795

[1] Concentration that reduces HCMV late antigen expression by 50%

Table S2. Effects of eltrombopag in combination with deferasirox and foscarnet. Combination indices (CIs) at different levels of inhibition and weighted average CI values (CI_{wt}) calculated as (CI_{50} + 2 × CI_{75} + 3 × CI_{90} + 4 × CI_{95}) / 10 [24] are provided. CI_{wt} values ≤ 0.7 indicate synergistic effects, CI_{wt} values > 0.7 and ≤ 0.9 moderately synergistic effects, CI_{wt} values > 0.9 and ≤ 1.2 additive effects, CI_{wt} values > 1.2 and ≤ 1.45 moderately antagonistic effects, and CI_{wt} values > 1.45 antagonistic effects [24]

	CI_{50}	CI_{75}	CI_{90}	CI_{95}	CI_{WT}	Combined effect
Eltrombopag +						
Deferasirox	4.22 ± 1.4	6.12 ± 1.2	9.03 ± 0.85	11.9 ± 1.7	9.11	antagonistic
Foscarnet	2.51 ± 0.35	1.1 ± 0.2	0.45 ± 0.1	0.25 ± 0.044	0.7	synergistic

Table S3. Effects of eltrombopag on HCMV late antigen (LA) expression in different cell types infected with different virus strains and isolates as determined 120h post infection. Concentrations that reduce LA expression by 50% (IC_{50}) or 90% (IC_{90}) are provided. The investigated eltrombopag concentrations did not affect cell viability. The eltrombopag concentration that reduced cell viability by 50% (CC_{50}) was >25,000nM for HFFs and 17,872 ± 1,302nM for ASCs.

	**Eltrombopag IC_{50} (nM)**	
**Virus strain**	**HFFs**	**ASCs**
Hi91	415 ± 197 (>60)[1]	4331 ± 1450 (4.1)
Davis	1182 ± 38 (>21)	265 ± 77 (67)
Towne	4230 ± 257 (>5.9)	2303 ± 616 (7.7)
U1	110 ± 7 (>227)	99 ± 30 (181)
U59	3372 ± 1253 (>7.4)	296 ± 84 (60)
U75	389 ± 93 (>64)	1828 ± 491 (9.8)

[1] Selectivity index (CC_{50}/ IC_{50})

	**Eltrombopag IC_{90} (nM)**	
**Virus strain**	**HFFs**	**ASCs**
Hi91	3379 ± 1088	9506 ± 2797
Davis	7086 ± 2796	9473 ± 1832
Towne	8683 ± 940	9697 ± 1428
U1	1621 ± 127	680 ± 129
U59	>10000	9237 ± 2051
U75	3578 ± 752	>10000
